# Assessment of Lumbar Intervertebral Disc Glycosaminoglycan Content by Gadolinium-Enhanced MRI before and after 21-Days of Head-Down-Tilt Bedrest

**DOI:** 10.1371/journal.pone.0112104

**Published:** 2014-11-07

**Authors:** Timmo Koy, Jochen Zange, Jörn Rittweger, Regina Pohle-Fröhlich, Matthias Hackenbroch, Peer Eysel, Bergita Ganse

**Affiliations:** 1 University of Cologne, Department of Orthopaedic and Trauma Surgery, Cologne, Germany; 2 German Aerospace Center (DLR), Institute of Aerospace Medicine, Department Space Physiology, Cologne, Germany; 3 Hochschule Niederrhein, Institute for Pattern Recognition, Krefeld, Germany; 4 University of Cologne, Department of Radiology, Cologne, Germany; 5 Cologne Center for Musculoskeletal Biomechanics (CCMB), Medical Faculty, University of Cologne, Cologne, Germany; 6 Department of Orthopaedic Trauma, RWTH Aachen University, Aachen, Germany; Medical University of Graz, Austria

## Abstract

During spaceflight, it has been shown that intervertebral discs (IVDs) increase in height, causing elongation of the spine up to several centimeters. Astronauts frequently report dull lower back pain that is most likely of discogenic origin and may result from IVD expansion. It is unknown whether disc volume solely increases by water influx, or if the content of glycosaminoglycans also changes in microgravity. Aim of this pilot study was to investigate effects of the spaceflight analog of bedrest on the glycosaminoglycan content of human lumbar IVDs. Five healthy, non-smoking, male human subjects of European descent were immobilized in 6° head-down-tilt bedrest for 21 days. Subjects remained in bed 24 h a day with at least one shoulder on the mattress. Magnetic Resonance Imaging (MRI) scans were taken according to the delayed gadolinium-enhanced magnetic resonance imaging (dGEMRIC) protocol before and after bedrest. The outcome measures were T_1_ and ΔT_1_. Scans were performed before and after administration of the contrast agent Gd-DOTA, and differences between T_1_-values of both scans (ΔT_1_) were computed. ΔT_1_ is the longitudinal relaxation time in the tissue and inversely related to the glycosaminoglycan-content. For data analysis, IVDs L1/2 to L4/5 were semi-automatically segmented. Zones were defined and analyzed separately. Results show a highly significant decrease in ΔT_1_ (p<0.001) after bedrest in all IVDs, and in all areas of the IVDs. The ΔT_1_-decrease was most prominent in the nucleus pulposus and in L4/5, and was expressed slightly more in the posterior than anterior IVD. Unexpected negative ΔT_1_-values were found in Pfirrmann-grade 2-discs after bedrest. Significantly lower T_1_ before contrast agent application was found after bedrest compared to before bedrest. According to the dGEMRIC-literature, the decrease in ΔT_1_ may be interpreted as an increase in glycosaminoglycans in healthy IVDs during bedrest. This interpretation seems contradictory to previous findings in IVD unloading.

## Introduction

Chronic lower back pain (CLBP) is a widespread disease in the population and also occurs in astronauts and cosmonauts [1], [2]. In many symptomatic cases, no structural cause is found in conventional MRI, and up to 85% of patients with CLBP do not have any visible anatomical anomalies [3]. Causes for back pain seem to be multifactorial [3], and degeneration of intervertebral discs (IVDs) is one known cause for discogenic pain [4], [5]. During spaceflight, IVDs gain height and cause elongation of the spine up to several centimeters [2], [6]. Astronauts frequently report moderate to severe, dull lower back pain that is most likely of discogenic origin and may result from IVD expansion [1], [6]. It is unknown whether the increase in disc volume is caused solely by water influx, or if the amount of glycosaminoglycans (GAGs) changes in microgravity [7], [8]. Furthermore, astronauts may have an increased risk for herniated nucleus pulposus, particularly in the immediate post-flight period [9]. Possible changes in IVD morphology are discussed as causing factors in the literature, however it is unknown what exactly happens within the IVD. The same type of back pain experienced in spaceflight was reported in bedrest studies with 6° head down tilt [10]. Bedrest studies have proven to be a good analog for changes in intervertebral disc morphology [7], [11]. Findings from space flight and bedrest might help to better understand the pathophysiology and treatment options for patients suffering from chronic lower back pain.

In its early stages, IVD degeneration involves a decrease in GAG content [12], [13]. While GAGs are known to decrease in IVD degeneration, an increase over a period of time in turn indicates recovery [14]. Standard MRI-imaging techniques are unable to detect early stages of IVD-degeneration [15], and so far microstructural changes and degeneration of lumbar intervertebral discs have not been assessed following space flight or bedrest.

A magnetic resonance imaging (MRI) method has been established that can quantify the loss of GAGs and detect degeneration (a decrease in GAG concentration) and recovery (an increase in GAG concentration). Called “delayed Gadolinium Enhanced MRI of Cartilage” (dGEMRIC), it was first applied to joint cartilage [16]–[19] and has recently been successfully utilized for the assessment of IVD degeneration [13], [20]–[22]. Standard MRI hardware is used for dGEMRIC imaging and measurements are performed before and after administration of a contrast agent that degrades and distributes in IVD tissue reciprocal to the amount of GAGs [12]. The longitudinal relaxation time (T_1_) in the tissue is shortened by the contrast agent. The effect intensity depends on the amount of contrast agent within the tissue. Thus changes in GAG-content of the IVD can indirectly be assessed through an analysis of T_1_-times in the MRI.

The aim of the present pilot study was to assess the GAG content of lumbar intervertebral discs before and after bedrest using the dGEMRIC protocol to investigate if the increase in IVD thickness during bed rest might be related to changes in GAG content. The hypothesis was that bedrest does not affect the amount of GAGs in the IVD. This hypothesis would be supported by the finding that the swelling of discs is accompanied by a decrease in GAG concentration. The alternative hypothesis was that the GAG concentration of intervertebral discs remains constant or increases during bedrest. Overall, the aim of the pilot study was achieved.

## Materials and Methods

### Ethics Statement

The study was approved by the ethics committee of North Rhine Medical Association (Ärztekammer Nordrhein, application number 2007405), and was designed and performed in compliance with the Declaration of Helsinki. Written informed consent was obtained from all subjects.

### Study setting

The experiment presented here was part of a large clinical trial (the NUC-Study), performed during one of the two campaigns, and it is by itself therefore not a clinical trial as defined in the CONSORT or TREND guidelines. The NUC-study was registered with the Clinical Trials Registry http://www.clinicaltrials.gov (Number: NCT01509456). It was also registered in the ESA Erasmus Experiment Archive http://eea.spaceflight.esa.int/portal/ (Experiment record no. 9389). The NUC-study took place in the Institute of Aerospace Medicine of the German Aerospace Center, Cologne, Germany.

### Study design

**Figure 1:**
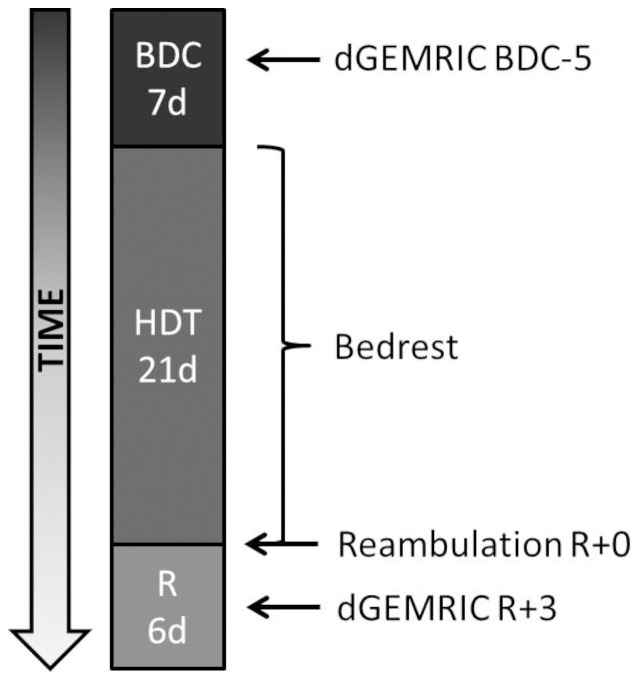
Time schedule of the study campaign. BDC = Baseline Data Collection, HDT = Head Down Tilt, R = Recovery. The MRI measurements presented here were performed five days before bedrest (pre-bedrest) and three days after (post-bedrest).

In the NUC-study, seven healthy, non-smoking, male human subjects were immobilized in 6° head-down-tilt bedrest for 21 days (HDT-1 to HDT-21) in a cross-over design. Five of these subjects were included in the presented investigation of IVDs. The entire bedrest-study included two campaigns for each subject, with a wash-out period of 154 days in between. The aim was to investigate a nutritional countermeasure for bone loss (oral application of potassium bicarbonate 30 mmol/tablet three times a day) in a cross-over design [7], [23], [24]. The dGEMRIC measurements presented here were conducted before and after the second campaign of the NUC-study beginning August 16^th^ and ending October 15^th^, 2010 (study schedule: Figure 1). For our experiment, it was anticipated that the nutritional countermeasure of the NUC-study would not have major effects on the formation of GAGs in the IVDs. Due to the small number of subjects, smaller potential effects could not have been found. During baseline and recovery data collection, subjects could move free inside the lab (baseline: 7 days, BDC-7 to BDC-1; recovery: 6 days, R+0 to R+5). Reambulation from bedrest took place in the morning of R+0. Throughout bedrest, subjects remained in bed 24 h/day with at least one shoulder on the mattress at any time. All hygienic procedures, food intake and experiments took place in this position without exception. Compliance with the protocol was ensured by video surveillance and by staff. Subjects did not undergo exercise or training. Psychological support was given by psychologists and medical doctors looked after the subjects in daily ward rounds. The dietary intake was strictly controlled by weighing all ingredients, food items and beverages for each test subject to prevent changes in bodyweight.

### Subject selection

Subject recruitment was announced on the institution's website as well as by flyers and posters in several universities and research institutes. Only male subjects were recruited for this study because the European Space Agency expected hormonal variations in female subjects to mimic effects in some of the experiments. Subject selection included a variety of clinical and laboratory tests as well as a psychological examination. Inclusion criteria were: male gender, age 20–45 years, body mass index 20–26 kg/m^2^, body height 158–190 cm (62–75 inches), body weight 65–85 kg and willingness to participate in the entire study. Exclusion criteria included abuse of drugs, medicine, nicotine or alcohol, regular medication, vegetarians and vegans, history of mental illness, rheumatic diseases, chronic hypertension, diabetes, obesity, arthritis, hyperlipidaemia, renal dysfunction, thyroid dysfunction, hepatic disease, disorders of calcium and bone metabolism, exercising more than four times a week, chronic back pain, a history of intervertebral disc prolapse, muscle and joint disease, family history of thrombosis and blood clotting disorders (Tests performed included AT III, Lupus-PTT, ferritin, Factors II, IV and V Leiden).

### The dGEMRIC protocol

**Figure 2:**
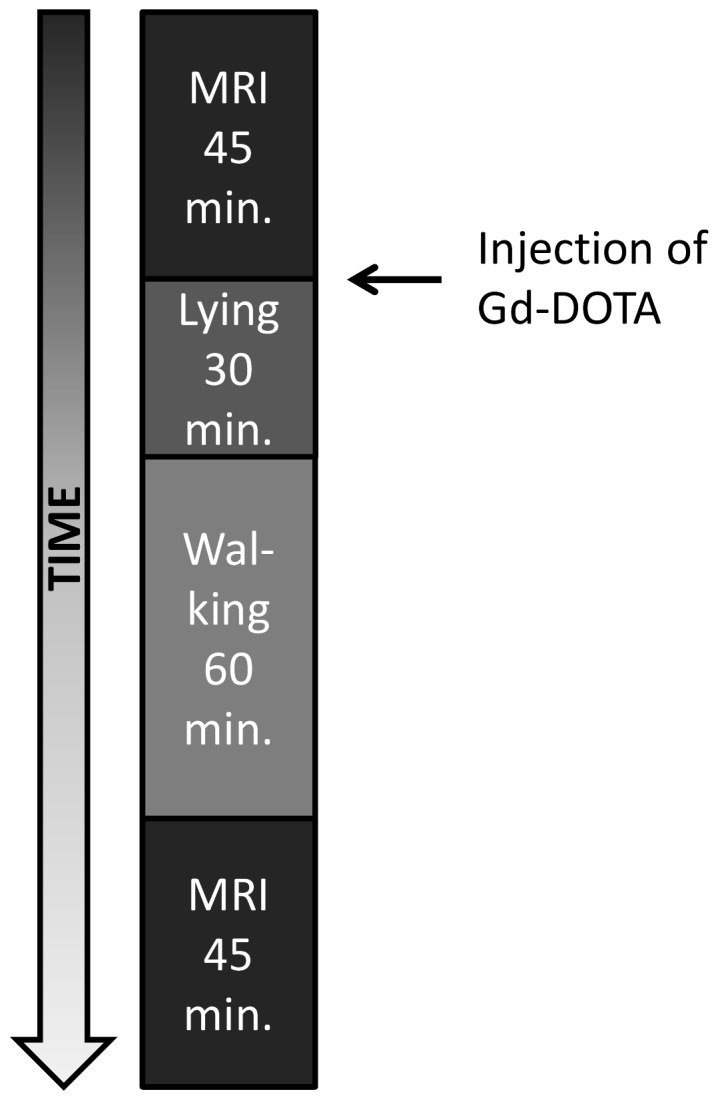
Protocol for dGEMRIC measurements.

MRI scans of the longitudinal relaxation time were taken according to the dGEMRIC protocol as described in the literature [14], [20]–[22]. Each subject underwent MRI scans of the lumbar spine pre- and post-bedrest on BDC-5 and R+3 (Figure 1). MRI scans were taken with a phased-array back coil in a 3T MRI scanner (Achieva, Phillips Medical Systems). A fast spin spin-echo inversion recovery sequence was applied which allows the pixel-wise calculation of T_1_ from a series of seven images recorded with fixed TR (1800 ms) and TE (13 ms) and variable inversion-recovery times (TI 50, 150, 350, 700, 1050, 1400 and 2000 ms, respectively). For each examined intervertebral disc, two series of cross-sectional images were individually planned based on sagittal pilot images of the spine. The images came from two consecutive slices recorded with 300 mm×300 mm field of view, an acquisition matrix of 512×512 pixels and 3 mm slice thickness. Each time, MRI scans were performed before and after i.v.-administration of the gadolinium-based contrast agent Gd-DOTA (Dotarem, Gadoteric acid, 0,4 mg/kg bodyweight). The schedule of Gd-DOTA administration, subject handling, and the measurements followed the protocol of Niikimäki et al. [20] and is shown in Figure 2.

### Calculation of T_1_-values

The automatic normalization of signal intensity was accidentally not disabled during some of the measurements before bedrest. In this case the signal intensity of the seven images in a series must be corrected prior to the calculation of T_1_. For the correction of signal intensities, a normalization-factor was calculated using manually chosen points in the subcutaneous fat tissue. T_1_-values of fat were known from the correctly recorded images and were proven to remain constant after the application of contrast agent. All post-bedrest measurements were performed without this technical error.

Finally, T_1_ was calculated for selected regions in the disc by fitting the signal intensities of selected pixels in the seven images and the corresponding inversion recovery times to the Nelder-Mead equation as published by Vaga et al. [21] using multidimensional unconstrained nonlinear minimization.

### Segmentation

**Figure 3:**
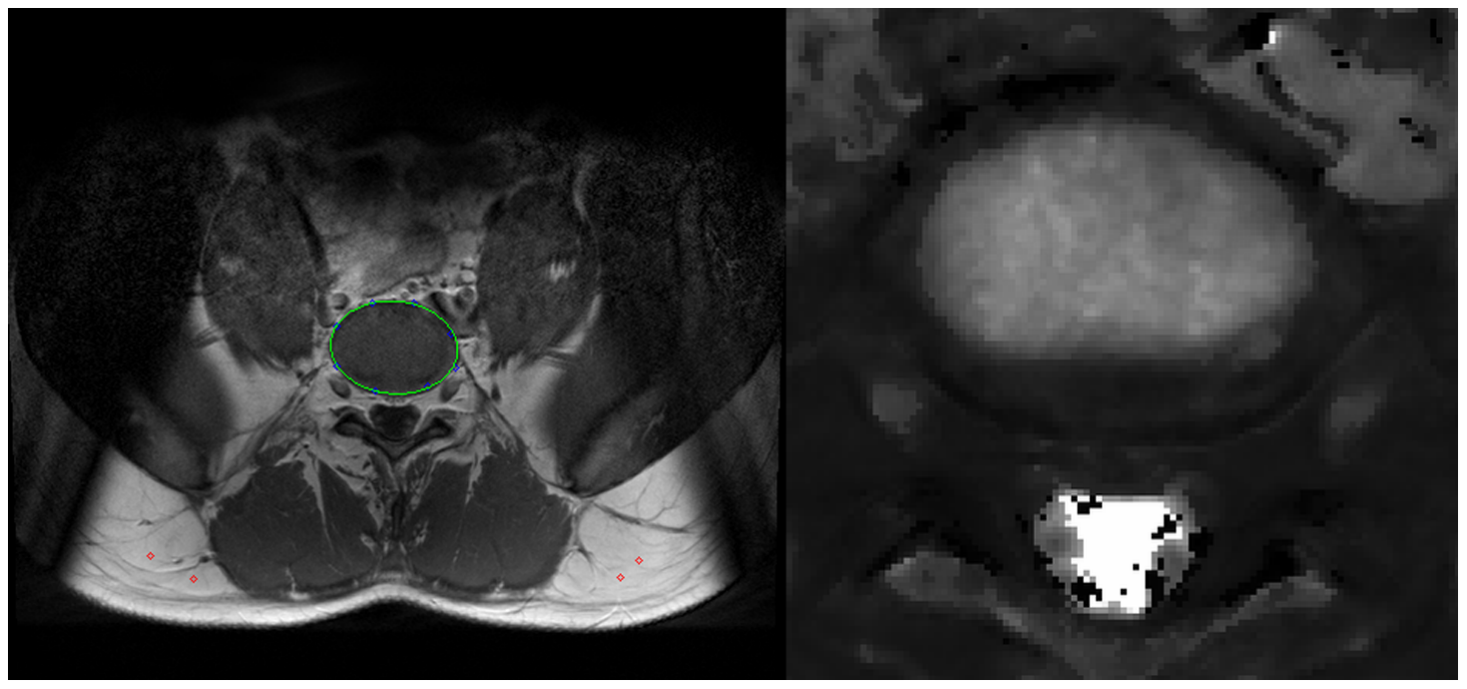
Selection of the intervertebral disc (left), calculated T1-map for subject D (right).

**Figure 4:**
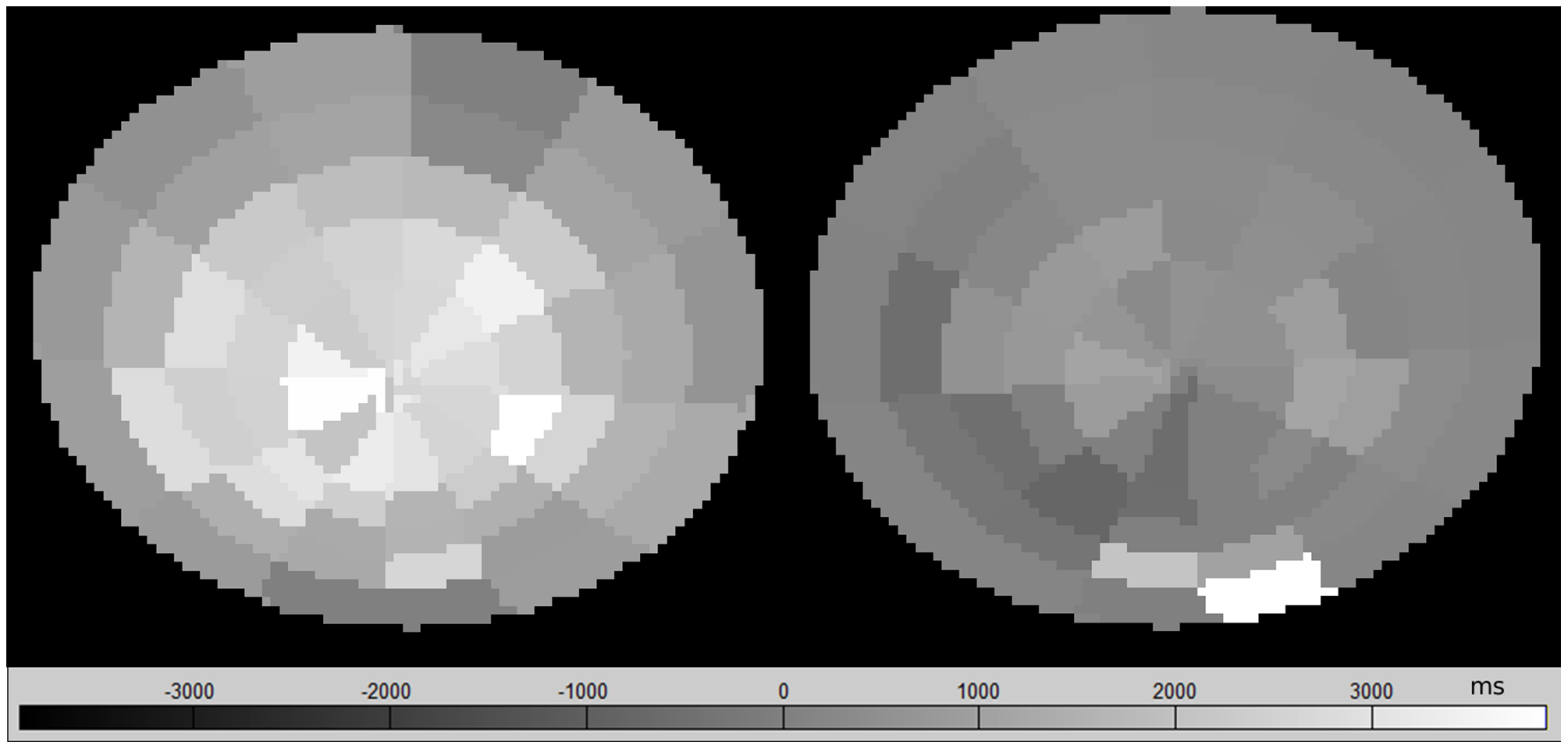
ΔT1-maps. Subject A, L1/2 divided into sectors, before (left) and after (right) bedrest.

**Figure 5:**
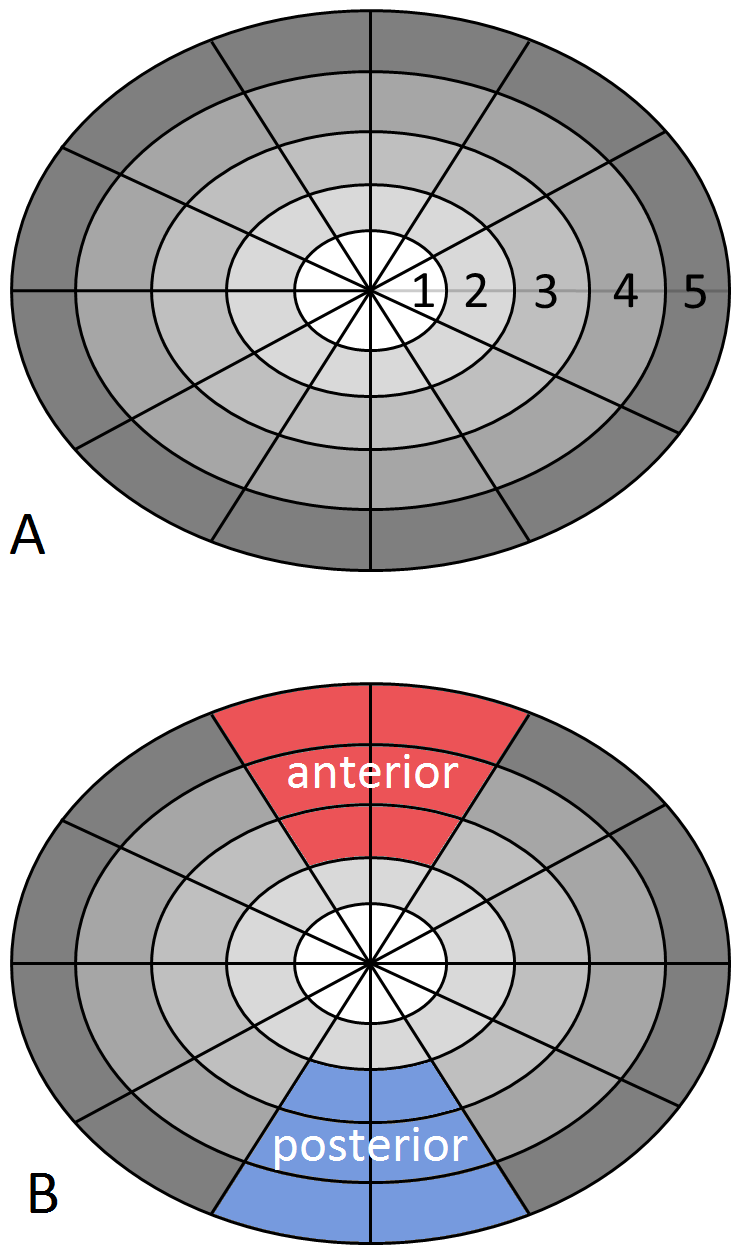
Segmentation for data analysis. Rings (A) and sectors of the annulus fibrosus (B) are shown. Rings 1 and 2 represent the nucleus pulposus and rings 3 to 5 correspond to the annulus fibrosus.

T_1_-maps were calculated for L1/2 to L4/5. The IVD region was segmented semiautomatically (Figure 3). An ellipse was fitted to eight points that were set manually. The ellipse was then subdivided into 60 sectors (Figure 4). For each sector, means and SD of the T_1_-times, as well as ΔT_1_ were computed. Rings and zones were defined and separately analyzed (Figure 5).

### Pfirrmann-grading

Sagittal T_2_ (spin-spin relaxation time) weighted multi echo images of the spine were acquired using a 1.5 T MRI scanner (Sonata, Siemens Medical Systems, Erlangen) with TR: 2500 ms, 15×TE: 10,3 ms–164,8 ms, 256×256 pixels in FOV: 330 mm×330 mm, 3 mm slice thickness, 6 mm interslice gap with a dedicated spine coil. The images were evaluated for Pfirrmann-grading of IVD degeneration by a radiologist [25]. Pfirrmann-grades were correlated with findings from dGEMRIC measurements.

### Statistical analysis

Using factorial ANOVAs (Statistica 10, Statsoft, Tulsa, OK, USA) native T_1_ values, T_1_-values after administration of the contrast agent, and ΔT_1_ were tested for group effects caused by bedrest, differences between regions within each disc and differences between different discs. Significance was assumed at p<0.05. Data are presented as counts and percentages, and as means and their sd. Exclusion conditions were T_1_ times <400 ms and >1500 ms. Where significance was found, a Tukey's post-hoc test was performed. SigmaPlot was used for plotting of data. ΔT_1_ was the primary outcome measure of this study.

## Results

Five out of seven subjects completed the entire experiment (Table 1). Two subjects were excluded from the analysis due to incomplete data sets (loss of data due to a software problem). There was no adverse event in connection with the dGEMRIC measurements.

**Table 1:** Details of the subjects.

Subject	Age (years)	Weight (kg)	Height (cm)
A	27	79.2	185
B	26	71.5	182
C	30	88.8	178
D	23	74.4	179
F	33	85.5	186
Mean (sd)	27.8 (3.8)	79.9 (7.3)	182 (3.5)

The main findings of this study were: 1. a decrease in ΔT_1_ after bedrest compared to before, 2. negative ΔT_1_-values, particularly in Pfirrmann-grade 2-discs after bedrest and in L4/5, and 3. significantly lower native T_1_ values after bedrest than before bedrest.

Average ΔT_1_ value of all intervertebral discs was 104.87 ms (sd 7.64 ms) pre-bedrest and -20.20 ms (sd 4.70 ms) post-bedrest. This difference is highly significant (p<0.001). Table 2 gives an overview of data, showing average values and standard deviations.

**Table 2:** Average values and standard deviations.

	Pre-contrast pre-bedrest	Pre-contrast post-bedrest	Post-contrast pre-bedrest	Post-contrast post-bedrest	Delta T1 pre-bedrest	Delta T1 post-bedrest
**L1/2**	934.41 (SD 19.54)	813.23 (SD 19.04)	784.66 (SD 17.03)	794.80 (SD 19.46)	149.76 (SD 15.83)	18.43 (SD 16.13)
**L2/3**	884.06 (SD 17.81)	774.13 (SD 15.55)	780.38 (SD 15.45)	796.16 (SD 15.75)	103.69 (SD 11.95)	−22.03 (SD 11.39)
**L3/4**	866.15 (SD 18.26)	788.02 (SD 17.01)	766.18 (SD 17.51)	805.20 (SD 18.60)	99.97 (SD 12.06)	−17.19 (SD 12.23)
**L4/5**	853.50 (SD 22.35)	766.44 (SD 18.35)	705.00 (SD 17.86)	814.75 (SD 16.69)	148.51 (SD 18.14)	−48.31 (SD 16.21)
**Ring 1**	1131.38 (SD 13.39)	972.09 (SD 11.92)	972.32 (SD 15.82)	992.94 (SD 11.13)	159.06 (SD 14.60)	−20.85 (SD 15.58)
**Ring 2**	1007.97 (SD 18.06)	872.82 (SD 15.69)	864.45 (SD 16.09)	904.37 (SD 15.36)	143.52 (SD 15.67)	−31.55 (SD 15.69)
**Ring 3**	916.16 (SD 18.17)	789.31 (SD 15.52)	756.98 (SD 14.33)	799.49 (SD 14.85)	159.18 (SD 17.11)	−10.19 (SD 14.68)
**Ring 4**	680.31 (SD 10.78)	591.37 (SD 11.06)	602.19 (SD 12.85)	603.88 (SD 11.77)	78.12 (SD 15.16)	−12.51 (SD 14.53)
**Ring 5**	532.66 (SD 10.27)	490.62 (SD 17.55)	495.44 (SD 10.24)	512.16 (SD 20.97)	37.22 (SD 13.17)	−21.54 (SD 15.12)
**Anterior sector**	560.13 (SD 20.66)	555.28 (SD 30.26)	537.03 (SD 19.11)	603.61 (SD 34.35)	23.10 (SD 24.41)	−48.33 (SD 36.22)
**Posterior sector**	855.94 (SD 30.70)	739.68 (SD 24.41)	686.21 (SD 24.02)	730.06 (SD 27.48)	169.73 (SD 27.68)	9.62 (SD 25.93)

### Differences between IVDs

**Figure 6:**
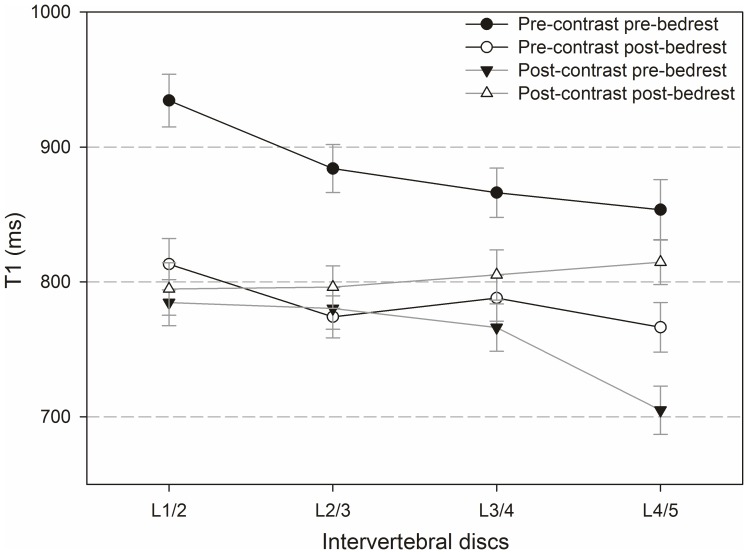
T_1_-values before and after administration of contrast agent, pre- and post bedrest for all intervertebral discs.

**Figure 7:**
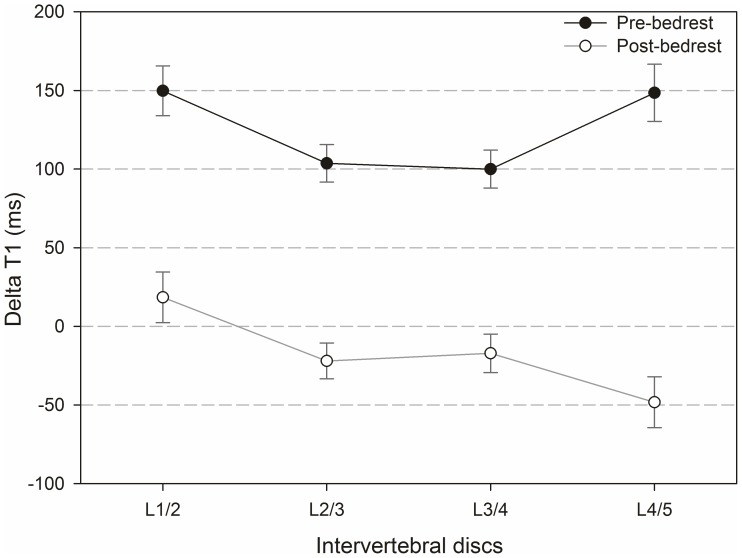
ΔT_1_-values before and after bedrest.

Figures 6 and 7 compare native and Gd-affected T_1_-values (Figure 6) and ΔT_1_-values (Figure 7) in different IVDs before and after bedrest.

Regarding native T_1_-values, no significant differences were found between IVDs comparing before and after bedrest. After the application of the contrast agent, a significant difference occurred between L1/2 and L4/5 pre-bedrest (p = 0.006), but there was no significant difference between IVDs post-bedrest. The effect of bedrest on pre-contrast T_1_ was significant for all discs (L1/2, L2/3 and L3/4: p<0.001 and L4/5: p = 0.021). The effect of bedrest on Gd-affected T_1_ was not significant for L1/2, L2/3 and L3/4, but for L4/5 (p = 0.002).

Before bedrest, positive ΔT_1_-values were found as anticipated effects of the contrast agent in all IVDs. Surprisingly, after bedrest, negative ΔT_1_-values were found in L2/3, L3/4 and L4/5. Negative ΔT_1_-values resulted from a longer T_1_ after the application of the contrast agent, which is physically incompatible with a mere effect of Gd uptake into the discs. In terms of the negative ΔT_1_ values found after bedrest, differences between IVDs were neither significant before nor after bedrest with one exceptional difference between L1/2 and L4/5 after bedrest (p = 0.049). The bedrest-induced decrease in ΔT_1_ was significant for all IVDs (p<0.001). Changes induced by bedrest were strongest in L4/5.

### Differences between inner and outer regions of the IVDs

**Figure 8:**
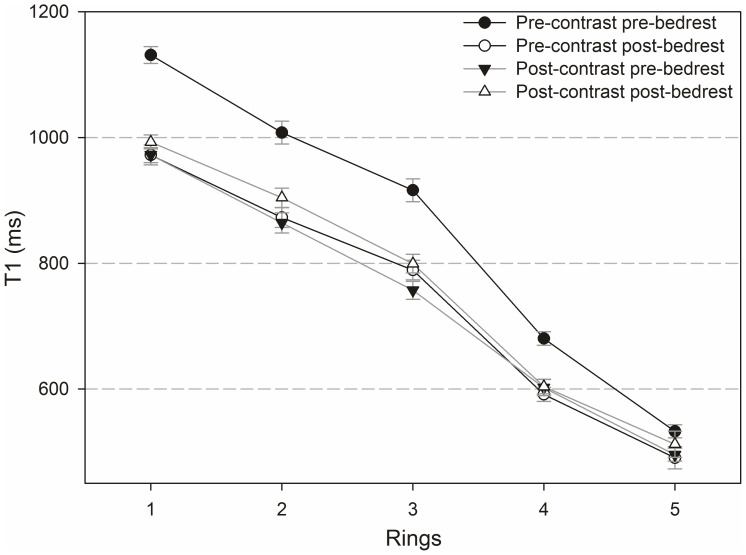
T_1_ values before and after administration of contrast agent, pre- and post-bedrest. Differences between rings according to segmentation as shown in Figure 5.

**Figure 9:**
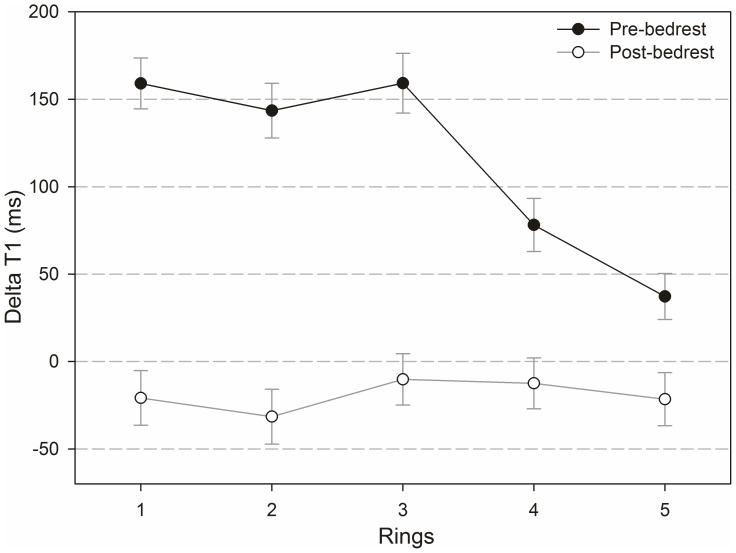
ΔT_1_-values pre- and post-bedrest plotted for each ring.

Figures 8 and 9 show T_1_-values pre- and post-contrast and pre- and post-bedrest (Figure 8) as well as ΔT_1_-values plotted for each selected ring pre- and post-bedrest (Figure 9). The central region was numbered as 1 and the following outer rings were numbered from 2 to 5. The nucleus pulposus of an IVD is covered by region 1 and ring 2, and the annulus fibrosus by rings 3 to 5.

Overall, T_1_-values were highest in region 1 and lowest in ring 5 with a continuous decrease from the centre of the IVD to the periphery. Differences in T_1_ between all rings were significant within each of the four categories (pre- and post Gd application, pre and post-bedrest) shown in Figure 8 (p<0.001; the only exception is the difference between rings 2 and 3 post-bedrest pre-contrast: p = 0.008). The bedrest-effect on T_1_-values pre-contrast is significant in all rings (rings 1–3 p<0.001 and ring 4 p = 0.024) except for ring 5.

Before bedrest ΔT_1_ was positive in all rings with an average of 133.23 ms (sd 7.48 ms). After bedrest ΔT_1_ was negative in all rings with an average of −19.73 ms (sd 7.33 ms). Before bedrest, differences of rings were significant between: rings 1 and 4 (p<0.001), rings 1 and 5 (p<0.001), rings 2 and 4 (p<0.001), rings 2 and 5 (p<0.001), rings 3 and 4 (p = 0.005) and rings 3 and 5 (p<0.001), but not between rings 1 to 3. Pre-bedrest ΔT_1_-values were higher in the nucleus pulposus than in the annulus fibrosus. Post-bedrest, there were no significant differences between rings. The bedrest-induced decrease in ΔT_1_ was significant in rings 1 to 4 (rings 1–3 p<0.001 and ring 4 p = 0.006), but not in ring 5.

### Findings in the anterior and posterior sector

**Figure 10:**
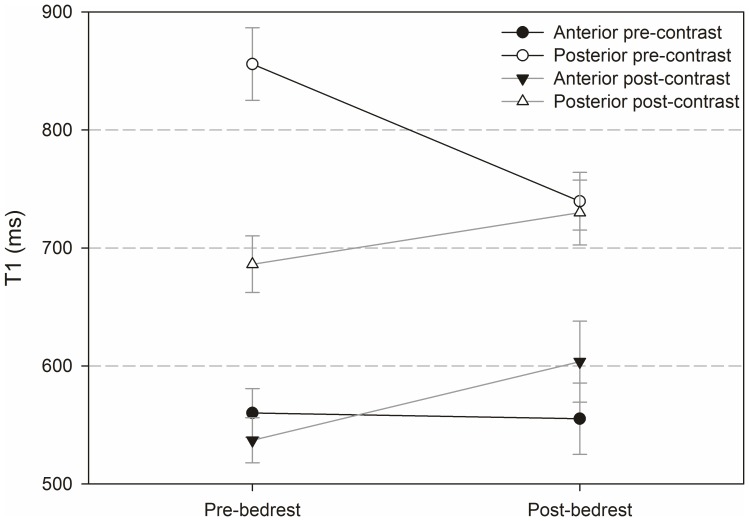
T_1_ values before and after administration of contrast agent, pre- and post-bedrest for the anterior and posterior segment as defined in Figure 4.

**Figure 11:**
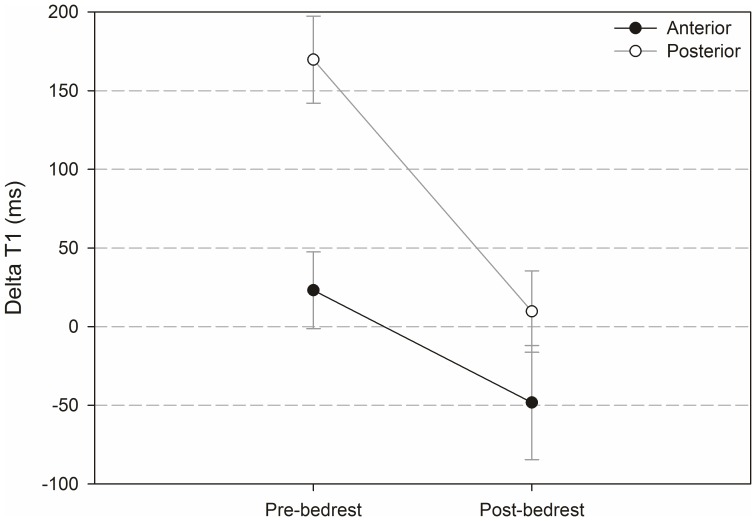
ΔT_1_-values pre- and post-bedrest for the anterior and posterior segment.

Figures 10 and 11 show results from analysis of the anterior and posterior segment of the intervertebral discs (segments are highlighted in Figure 5). The analysis includes T_1_-values pre- and post-contrast pre- and post-bedrest (Figure 10) and ΔT_1_-values (Figure 11).

The difference between anterior and posterior T_1_-values pre-contrast was significant before (p<0.001) and after (p = 0.024) bedrest. Post-contrast, it was only significant before bedrest (p = 0.029). The effect of bedrest was neither significant for anterior nor for posterior T_1_-values.

ΔT1 was significantly higher in the posterior sector compared to the anterior sector pre-bedrest (p<0.001) but not post-bedrest (p = 0.500). The difference between ΔT_1_ pre- compared to post-bedrest was significant in the posterior (P = 0.004) but not in the anterior segment.

### Pfirrmann-grading

Pfirrmann-grades of IVDs are shown in Table 3. Intervertebral discs with a Pfirrmann-grade >2 were excluded from the following analysis because there was only one case each. Statistical analysis reveals a significant difference between Pfirrmann-grade and ΔT_1_ (p = 0.006), but not between Pfirrmann-grade and T_1_ (p = 0.125). ΔT_1_ post-bedrest is 12.42 (sd 10.36) ms in Pfirrmann-grade 1 and −27.94 (sd 10.32) ms Pfirrmann-grade 2. Therefore the unexpected occurrence of negative ΔT_1_ post-bedrest corresponds with disc degeneration.

**Table 3:** Pfirrmann-grades.

Subject	L1/2	L2/3	L3/4	L4/5
A	2	2	1	1
B	2	1	1	1
C	2	2	2	2
D	4	2	3	2
F	2	2	1	2

There were no changes in values throughout bedrest.

## Discussion

The aim of this pilot study was to indirectly assess the GAG content of the lumbar intervertebral discs L1/2 to L4/5 before and after 21 days of bedrest using the dGEMRIC protocol to investigate if changes can be found. Results showed

1. A highly significant decrease in ΔT_1_ induced by the bedrest-intervention in L1/2 to L4/5, and in all areas of the IVD, that might be interpreted as an increase in GAGs in healthy IVDs during bedrest. The ΔT_1_-decrease was most pronounced in the nucleus pulposus and in L4/5 and was expressed slightly more in the posterior IVD.

2. Unexpected negative ΔT_1_-values were found in Pfirrmann-grade 2-discs after bedrest and in L4/5.

3. Significantly lower T_1_ before contrast agent application after bedrest compared to before bedrest.

The dGEMRIC protocol is a reliable method to measure changes in GAG-content of cartilage and IVDs [13], [16]–[22]. It has been used and validated in a number of clinical and experimental studies. It seems to be well established that increased GAG concentration within the IVD will result in a decrease in ΔT_1_ [13], [14], [20], [21]. A high GAG-concentration causes a small ΔT_1_ during dGEMRIC-measurements because only small amounts of contrast agent shift into the IVD [12]. A low GAG-concentration in turn leads to a high ΔT_1_. Increased ΔT_1_ after an intervention (as compared to before) has been interpreted as degeneration process in the literature [13], [14].

This study showed a decrease in ΔT_1_ after bedrest in the healthy lumbar IVDs (L1/2 to L4/5, Pfirrmann-grade 1), which, according to the literature, might be interpreted as GAG-increase [13], [14]. After bedrest, T_1_ was already decreased before the administration of contrast agent compared to before bedrest. This finding indicates that the intervention of bedrest had an effect on the IVDs. A theoretical possibility is that contrast agent might have remained within the IVD during bedrest and therefore caused decreased T_1_ after bedrest. Gd-DOTA, however, is excreted rapidly through the kidneys and concentrations in IVDs show their maximum 210 minutes after injection followed by a speedy decrease as shown by Vaga et al. [21]. As subjects walked normally for 4.5 more days after the injection of the contrast agent and before bedrest, remaining contrast agent in the IVD is very unlikely to explain the particularly short T_1_-times post-bedrest.

Surprisingly, ΔT_1_ showed negative values after bedrest in the IVDs with first signs of degeneration (Pfirrmann grade 2). This phenomenon, to our knowledge, has not been previously reported in studies using the dGEMRIC protocol to determine GAG-content of IVDs [13], [14], [20], [21]. It might be an incidental finding related to the small sample size. In this study, negative ΔT_1_-values result from an increase in T_1_ after injection of the contrast agent post-bedrest. This finding cannot be explained by an increase in GAG-content only. As the contrast agent shortens T_1_-time, mere increase in GAG-content would not cause longer T_1_ after Gd-DOTA administration. In case no contrast agent reaches the IVD, T_1_ should remain unchanged, but there is no way for it to increase just by contrast agent. It can neither be explained by disc degeneration, because a low amount of GAGs results in small ΔT1-values, but not in negative ΔT1-values. Therefore, an additional effect might have influenced our findings and led to the increase in T_1_ in the slightly degenerated IVDs after bedrest. Considering the contrast agent's chemistry, the Gd-DOTA-complex, due to its inertness is unlikely to interact with the intervertebral disc in a way that might alter T_1_-time. As free water shows longest T_1_, an increase in free water within the IVD might be a possible cause. The contrast agent, however, was injected between the two MRI measurements; subjects remained in supine position for 30 minutes and walked around for 60 minutes (Figure 2). In theory, compression of IVDs during walking would decrease the water content and not increase it [26]. Post-bedrest dGEMRIC-measurements were performed in the morning three days after bedrest. During these three days, subjects were already allowed to walk around while having a number of experiments (spiroergometry, DEXA, pQCT, different MRI measurements, muscle fatigue, eye examinations and ultrasound measurements). However, most of this time was not spent in the upright position, but rather sitting and lying. Therefore, walking for 60 minutes may have changed the composition of the already slightly degenerated IVDs. Processes such as osmosis or a pump mechanism might play a role here, e.g. by changing the content of free water by releasing bound water. Furthermore, it is unclear in how far intra-nuclear fissures and clefts might affect results in disc degeneration as well [27].

Regarding the negative ΔT_1_ values, it is thought that there is a fluid effect induced by degeneration processes, the number of subjects is too small and the finding is accidental, or the method dGEMRIC reveals its weaknesses in accuracy. The question how bedrest affects the GAG-content of degenerated discs in higher stages of degeneration needs to be addressed in future studies.

The results of this study are in accordance with results from Vaga et al. [14] and Ciavarro et al. [13] who both showed a GAG-increase in operatively stabilized lumbar IVDs. Contradictory findings were published by Hutton et al. [28] who found a significant decrease in proteoglycan content of IVDs in rats after four weeks of tail suspension as model for simulated weightlessness. It is however unclear how well IVDs are unloaded during tail suspension. A decrease in proteoglycan concentration was also found in rat-IVDs after 5 days of spaceflight [29]. Results of changes in GAG-content in human IVDs during simulated or actual spaceflight have not been published before. Comparability between species seems to be limited due to differences in cell cytomorphology [30], and biomechanical forces and strains differ between vertical and horizontal spines.

Vaga et al. [21] correlated the biochemistry-derived sGAG-content of IVDs and ΔT_1_ assessed by dGEMRIC, performed a linear regression analysis and found a regression function (y = −1,38x+238). Applying this regression function to the average post-bedrest-ΔT_1_ of Pfirrmann grade 1 discs found in the present study, a GAG-content of over 250µg/mg is found. This value may be slightly overestimated as Vaga et al. waited for 210 minutes instead of 90 minutes for the second MRI after injection of contrast agent, which would influence T_1_ by about 50 ms as shown in the same paper. In any way, the GAG-concentration resulting from ΔT1-values found in this study probably exceeds results from healthy IVDs published in the literature [21].

Regarding CLBP, our study results are in accordance with findings from Arvinen et al. [31] who have found out that an insufficient quantity of sleep is a risk factor for low back pain. Sufficient time in bed might be necessary for the IVDs to recover from the mechanical load and strain of the daily activities. Further studies are required to examine a possible connection between the daily time spent in bed and the IVDs GAG content, as well as CLBP incidence. In addition, further research on changes in composition of IVDs during bedrest needs to be conducted. Though results are highly significant, the present pilot study was performed on a small number of healthy subjects only, and results should be confirmed in a larger cohort and with different approaches.
